# Supplementary material for: Quantitative in vivo whole genome motility screen reveals novel therapeutic targets to block cancer metastasis
Source: Nat Commun. 2018 Jun 14;9:2343. doi: 10.1038/s41467-018-04743-2 (PMC6002534; doi:10.1038/s41467-018-04743-2)
Supplement: Supplementary file 1 — Supplementary Information [file 41467_2018_4743_MOESM1_ESM.pdf]

Quantitative *in vivo* whole genome motility screen reveals novel targets to block cancer metastasis.

Stoletov et al.

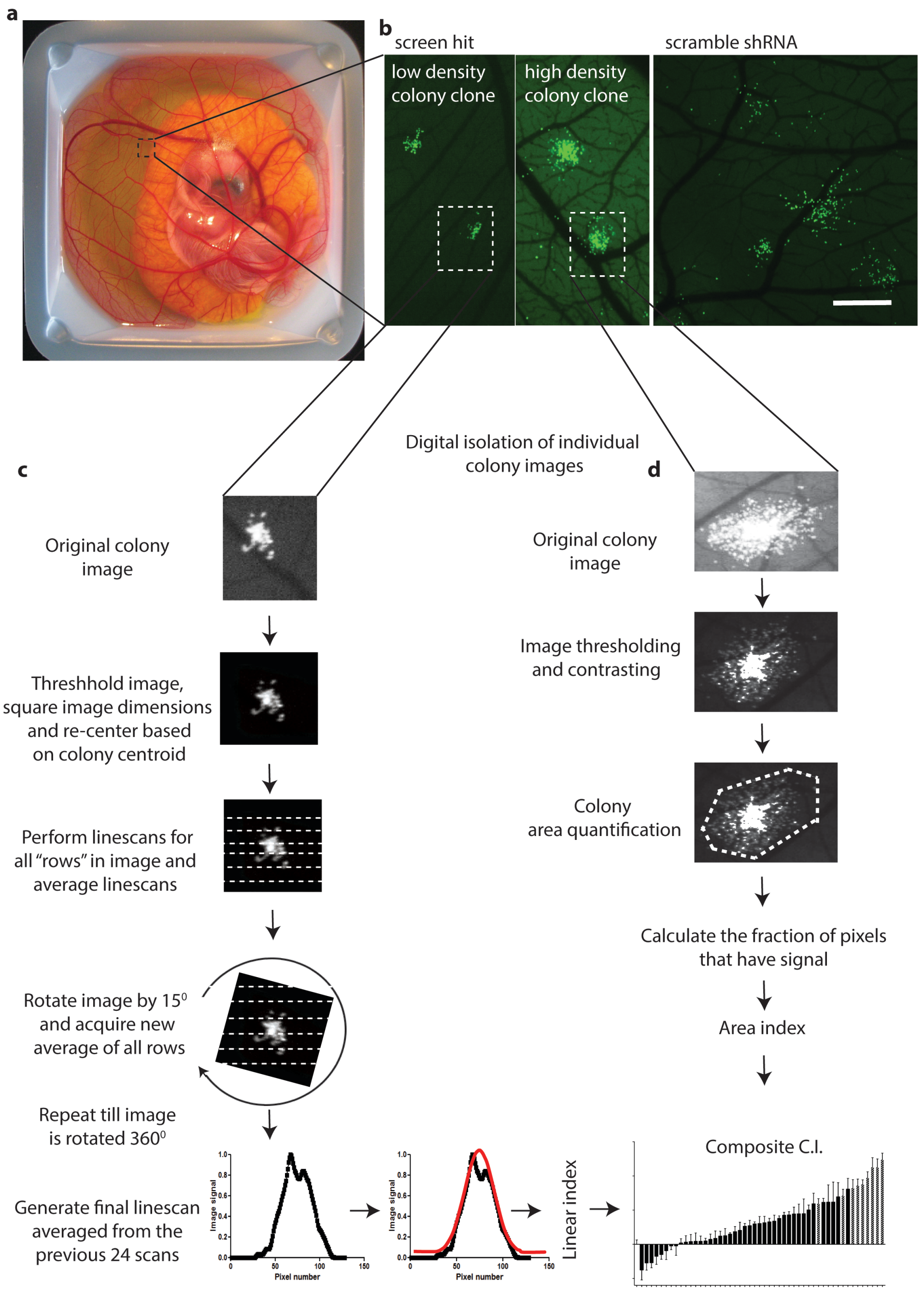

Supplementary Figure 1. Quantitative avian embryo *in vivo* screening platform. (**a**) Chicken embryo, 16 days post fertilization, in *ex ovo* culture. Dashed red circles in the top panels represent individual colony examples used for C.I quantification. (**b**) Representative images of metastatic colonies arising from single cancer cells, 6 days after intravenous injection. Left panel shows a colony derived from one of the screen hits (shSRPK1), right panel shows control, scramble shRNA transduced HEp3 colonies. **(c)** Schematic of the Linear Index metastatic colony compactness quantification approach. **(d)** Schematic of the Area and Density Index metastatic colony compactness quantification approaches. These three measures are combined for each screen hit to generate a composite Compactness Index (C.I.). Statistical significance was determined using one-way ANOVA with Fisher's LSD test (* p<0.05, ** p<0.01, *** p<0.001). Scale bar = 300μm.

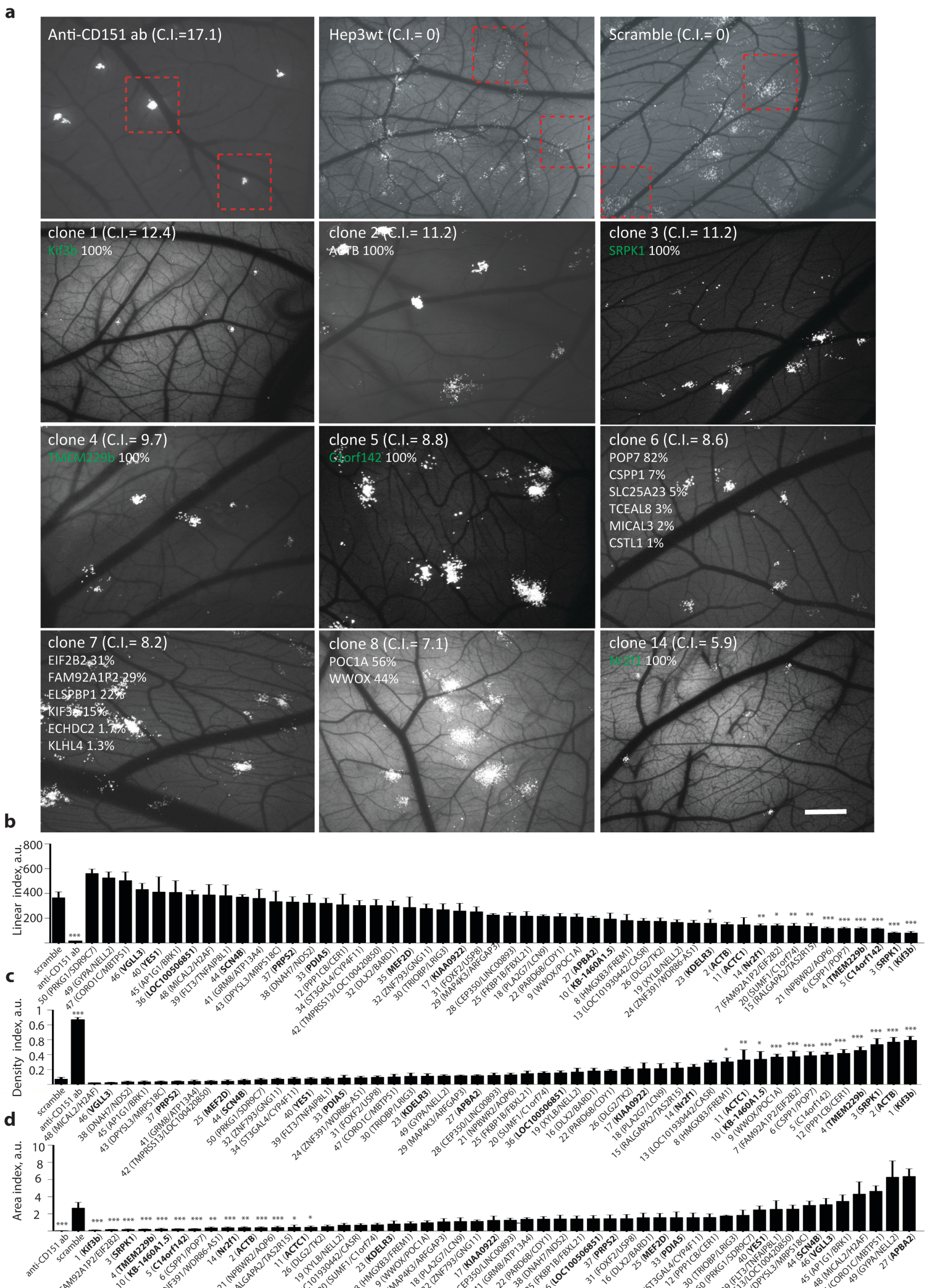

Supplementary Figure 2. Validation and quantification of *in vivo* motility phenotypes. (a) Representative images of shRNA screen clones that reproducibly formed compact colonies derived from single cells in the avian embryo CAM. Overlays show the composite C.I. scores and shRNAs present in the clone, sorted by their abundance. Representative colonies formed by original (wt) and scramble shRNA transduced HEp3 cells are also shown. shRNAs selected for further analysis are highlighted in green. Examples of digitally cut out colonies used for C.I. quantification are highlighted with red dashed squares. **(b)** Linear Index distribution of clones identified in the screen. **(c)** Density Index distribution of clones identified in the screen. **(d)** Area Index distribution of clones identified in the screen. Statistical significance was determined using one-way ANOVA with Fisher's LSD test (* p<0.05, ** p<0.01, *** p<0.001). Scale bar = 500µm.

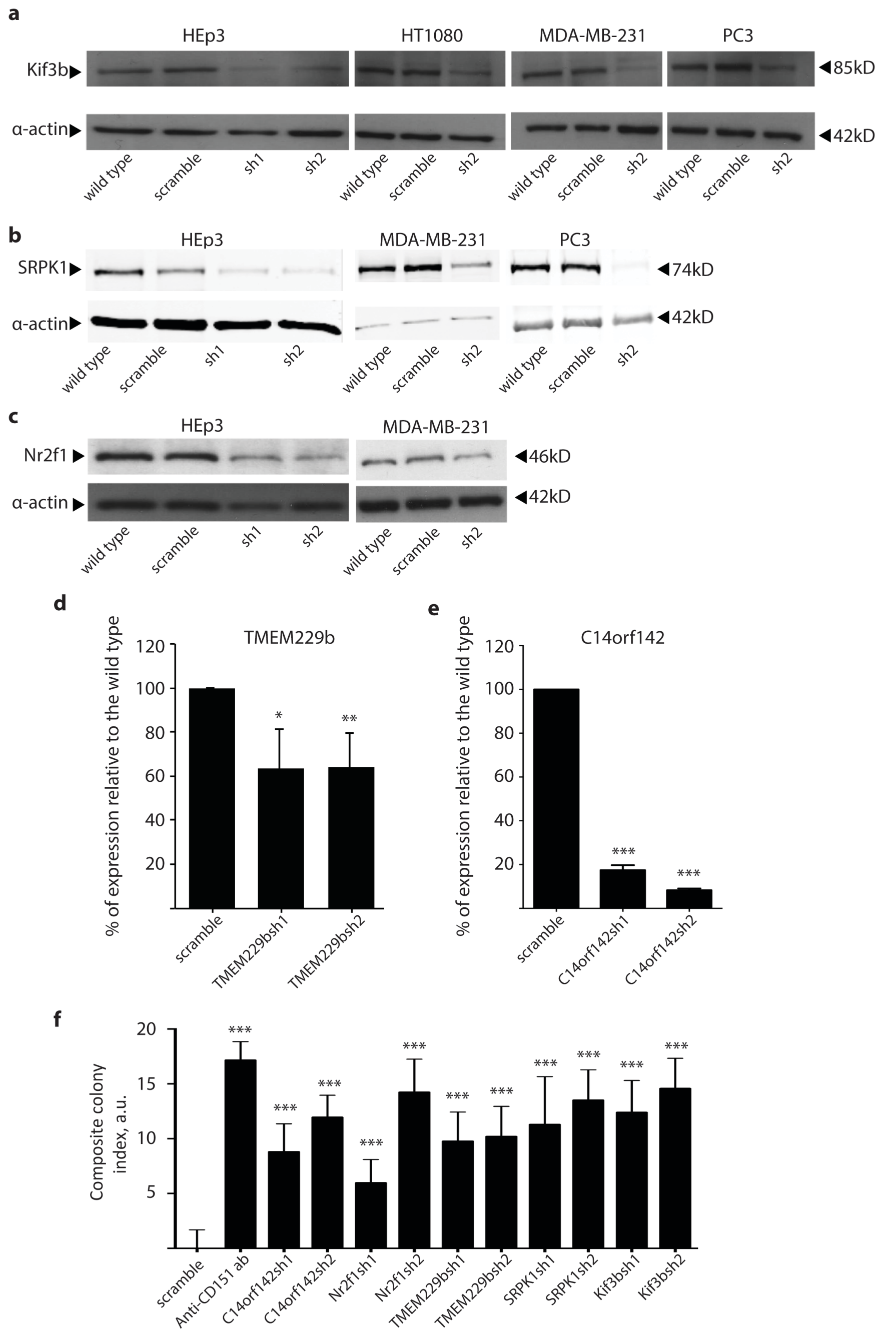

Supplementary Figure 3. Generation of a panel of cancer cell lines with stable knockdown of screen identified genes. (**a**) Western blot of KIF3Bsh1/sh2 and scramble shRNA control cell lines (HEp3, PC3 and MDA-MB-231). (**b**) Western blot of SRPK1sh/sh2 and scramble shRNA control cell lines (HEp3, PC3 and MDA-MB-231). (**c**) Western blot of NR2F1sh/sh2 and scramble shRNA control cell lines (HEp3 and MDA-MB-231). (**d**) qRT-PCR analysis of TMEM229Bsh/sh2 and scramble shRNA control cell lines (HEp3, normalized to control). **e)** qRT-PCR analysis of shC14orf142sh/sh2 and scramble shRNA control cell lines (HEp3, normalized to control). f) Composite compactness indexes (C.I.) for independent shRNA (sh2) clones. C.I. indexes for initial, screen selected clones (sh1) used as comparison. Statistical significance was determined using one-way ANOVA with Fisher's LSD test (* p<0.05, ** p<0.01, *** p<0.001).

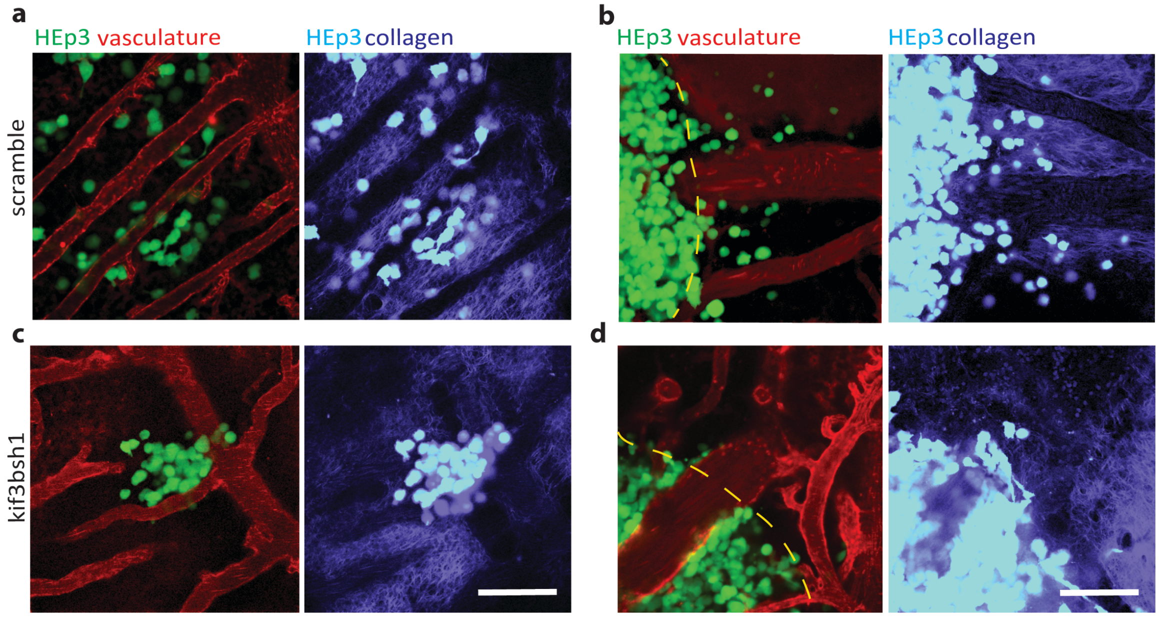

Supplementary Figure 4. Screen-isolated HEp3 clone that expresses anti-Kif3B shRNA (sh1) shows decreased vasculotropism and interaction with CAM collagen fiber network. Representative images showing metastatic colonies (**a**, **c**) and primary tumor fronts (**b**, **d**) formed by scramble or Kif3b sh1transduced HEp3 cells. Note that control cells robustly interact with the vasculature and invade into the collagen matrix while shRNA1 Kif3b cells fail to do so. Scale bars = 100µm.

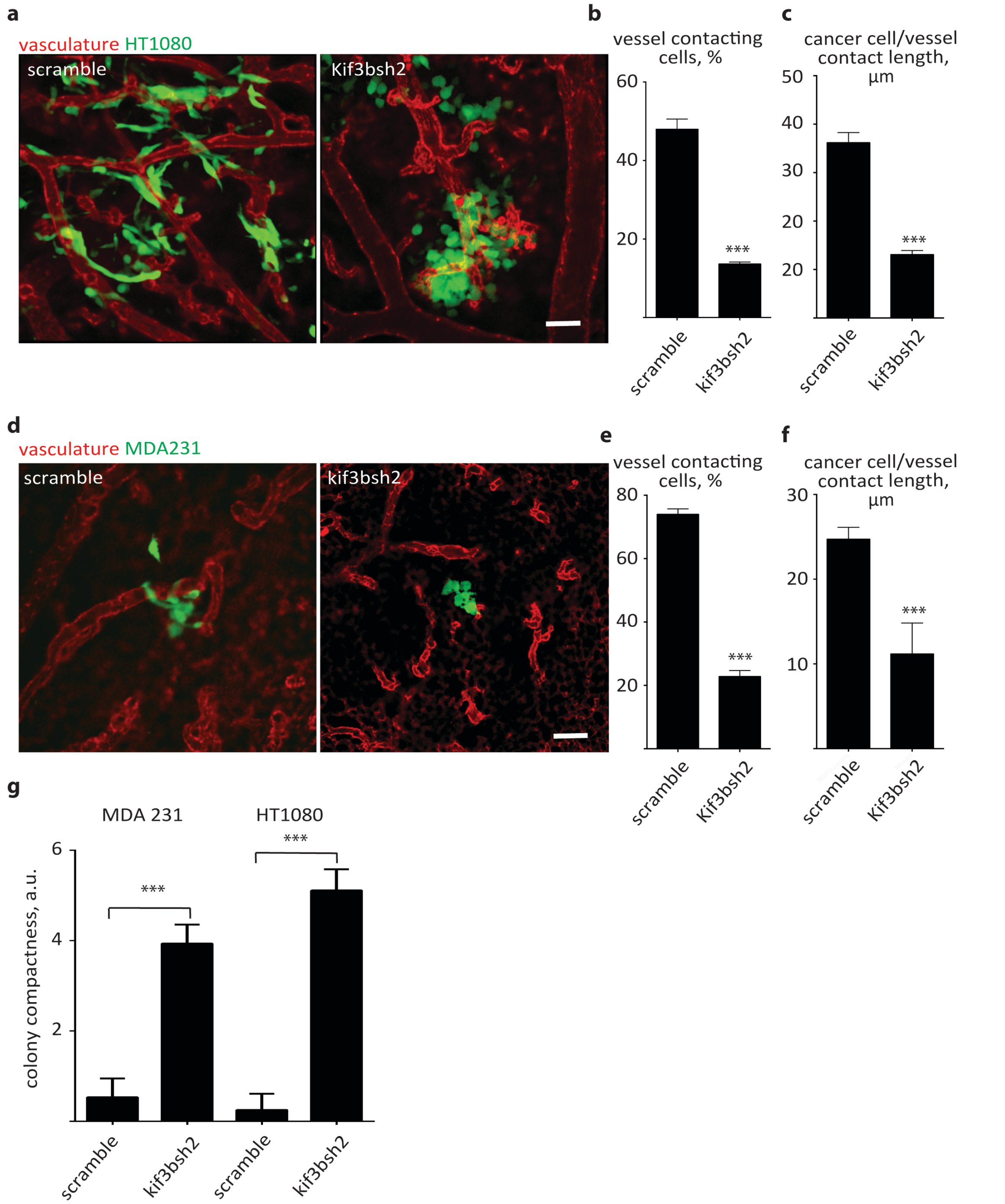

Supplementary Figure 5. Inhibition of KIF3B expression in HT1080 and MDA231 leads to compact colony phenotype. (a) Representative images showing metastatic colonies formed by scramble and Kif3b sh2 transduced HT1080 cells. Quantification of percentage of vessel contacting cells (**b**) and cell/vessel contact lengths (**c**) for metastatic colonies formed by scramble and Kif3b sh2 transduced HT1080. (**d**) Representative images showing metastatic colonies formed by scramble and Kif3b sh2 transduced MDA231 cells. Quantification of percentage of vessel contacting cells (**e**) and cell/vessel contact lengths (**f**) for metastatic colonies formed by scramble and Kif3b sh2 transduced MDA231 cells. (**g**) Quantification of C.I. index for metastatic colonies formed by scramble and Kif3b sh2 transduced HT1080 and MDA231 cells. Statistical significance was determined using unpaired t -tests (*** = $p<0.001$). Scale bars = 100µm.

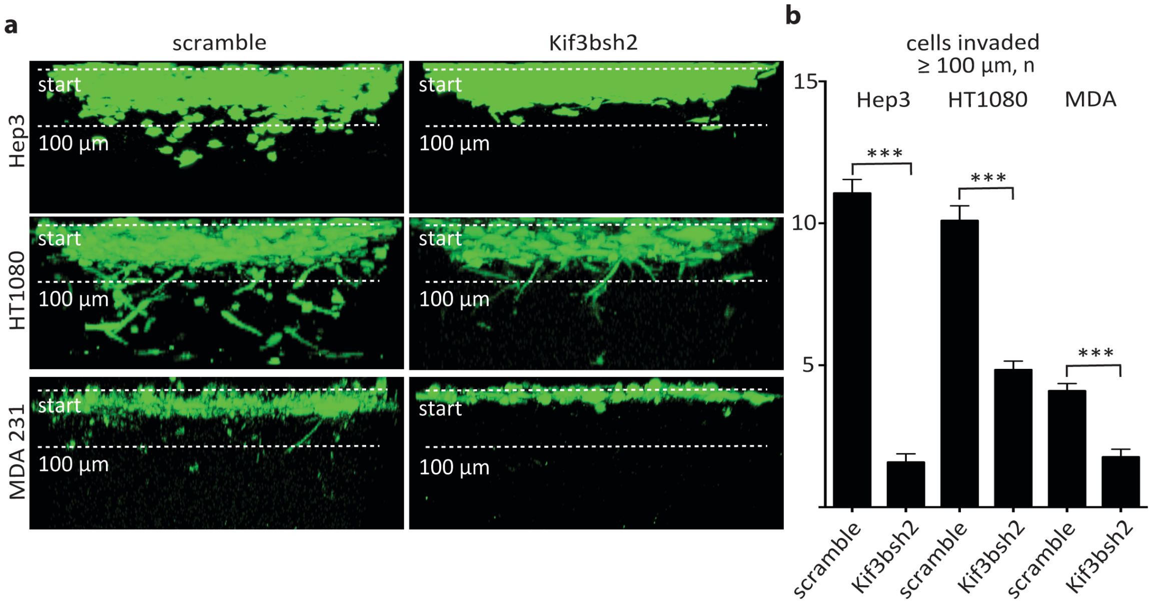

Supplementary Figure 6. Effect of KIF3B inhibition on *in vitro* 3D cancer cell invasion. a) Representative 3D confocal image stacks (side view) showing relative invasion of HEp3, HT1080 and MDA231 cancer cells that were transduced with either control, scramble shRNA or Kif3b targeting shRNA (sh2). (**b**) Quantification of 3D collagen gel invasion for HEp3, HT1080, MDA231 cancer cells and their Kif3b sh2 transduced variants. Statistical significance was determined using unpaired t -tests (*** = p<0.001).

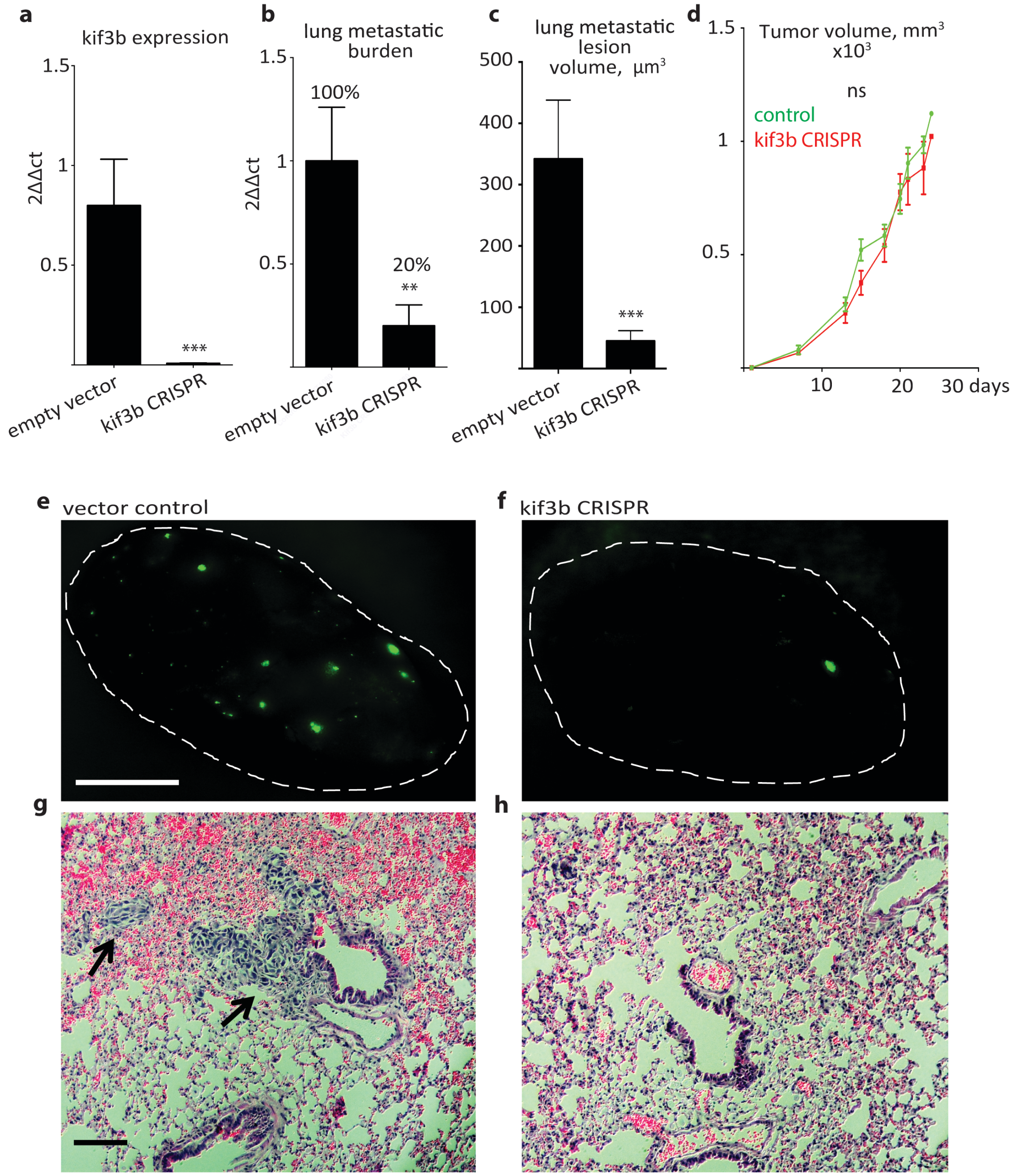

Supplementary Figure 7. CRISPR-mediated Kif3b gene deletion blocks spontaneous metastasis of HT1080 cancer cells *in vivo*. (**a**) Q-PCR quantification of Kif3b expression in control (empty vector) and Kif3b CRISPR knock out cells. (**b**) q-PCR quantification HEp3 cancer cells metastasized to lung as determined by human *alu* q-PCR. (**c**) Primary tumor weight at the time of sacrifice for the mice inoculated with control or Kif3b knockout HT1080 cells. (**d**) Volume of lung metastatic lesions for mice inoculated with Kif3b knockout HT1080 cells. Fluorescence stereomicroscopic images of lungs from mice bearing subcutaneous tumors derived from HT1080 cancer cells transduced with (**e**) control empty vector, (**f**) CRISPR construct targeting KIF3B; dashed line delineates the organ borders. (**g, h**) Representative IHC (H&E) fields of mouse lungs from mice in (**e**) and (**f**); Arrows show the HT1080 cancer cell metastatic lesions within the lung. . Statistical significance was determined using unpaired t -tests (** p<0.01, *** = p<0.001). Scale bars = 1mm (**e**, **f**) or 100µm (**g**, **h**).

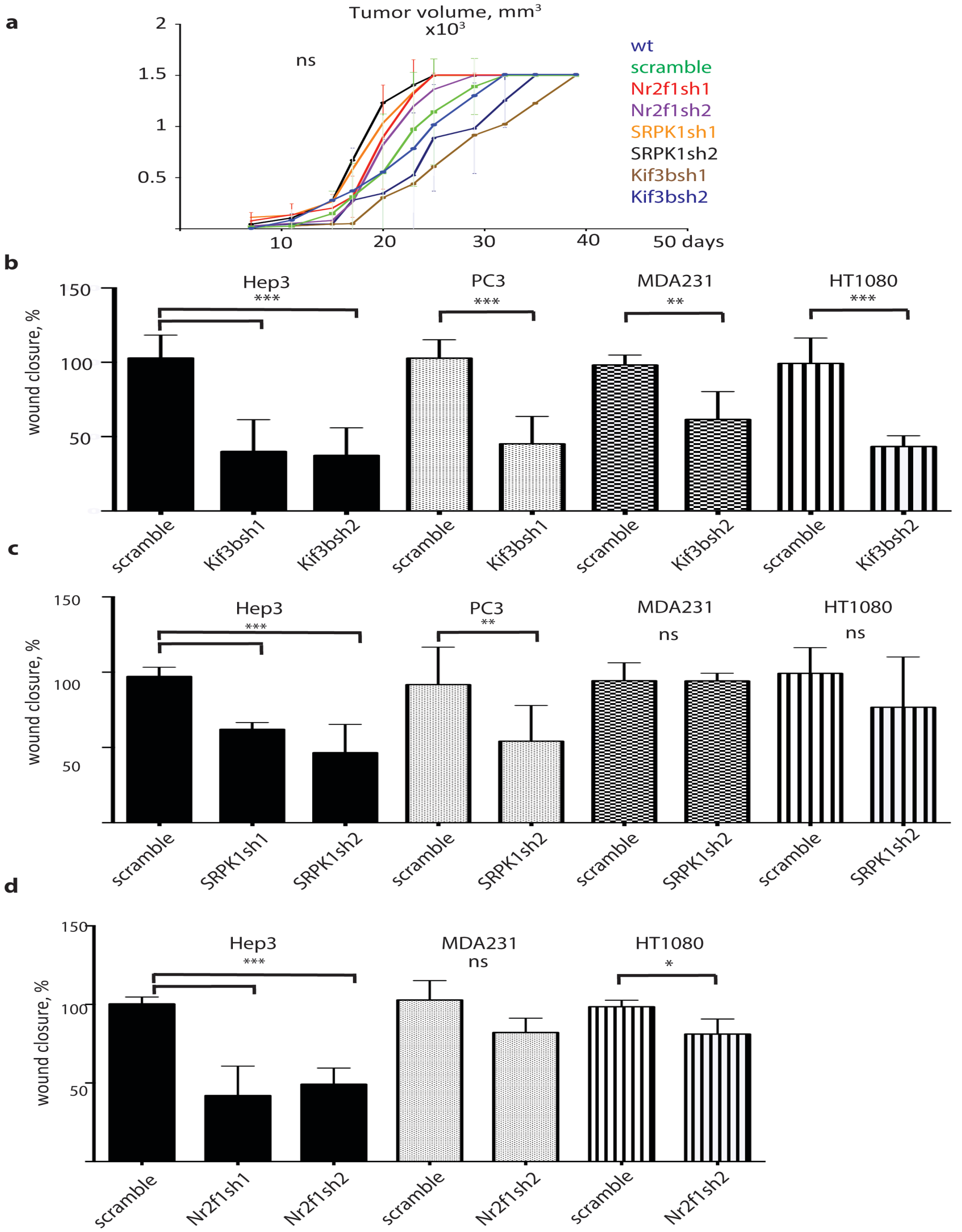

Supplementary Figure 8. Effect of KIF3B, SRPK1 and NR2F1 inhibition on *in vitro* cancer cell migration. (a) Primary tumor weight of control and knockdown cell lines induced tumors used in the experiment. **(b)** MAts (magnetically attachable stencils) *in vitro* migration assay of control and KIF3Bsh1/sh2 cell lines[30]. **(c)** MAts *in vitro* migration assay of control and mutant SRPK1sh1/sh2 cell lines. (**d**) MAts *in vitro* migration assay of control and NR2F1sh1/sh2 cell lines. Values are normalized to the scramble shRNA control. Statistical significance was determined using unpaired t -tests (* p<0.05, ** p<0.01, *** p<0.001).

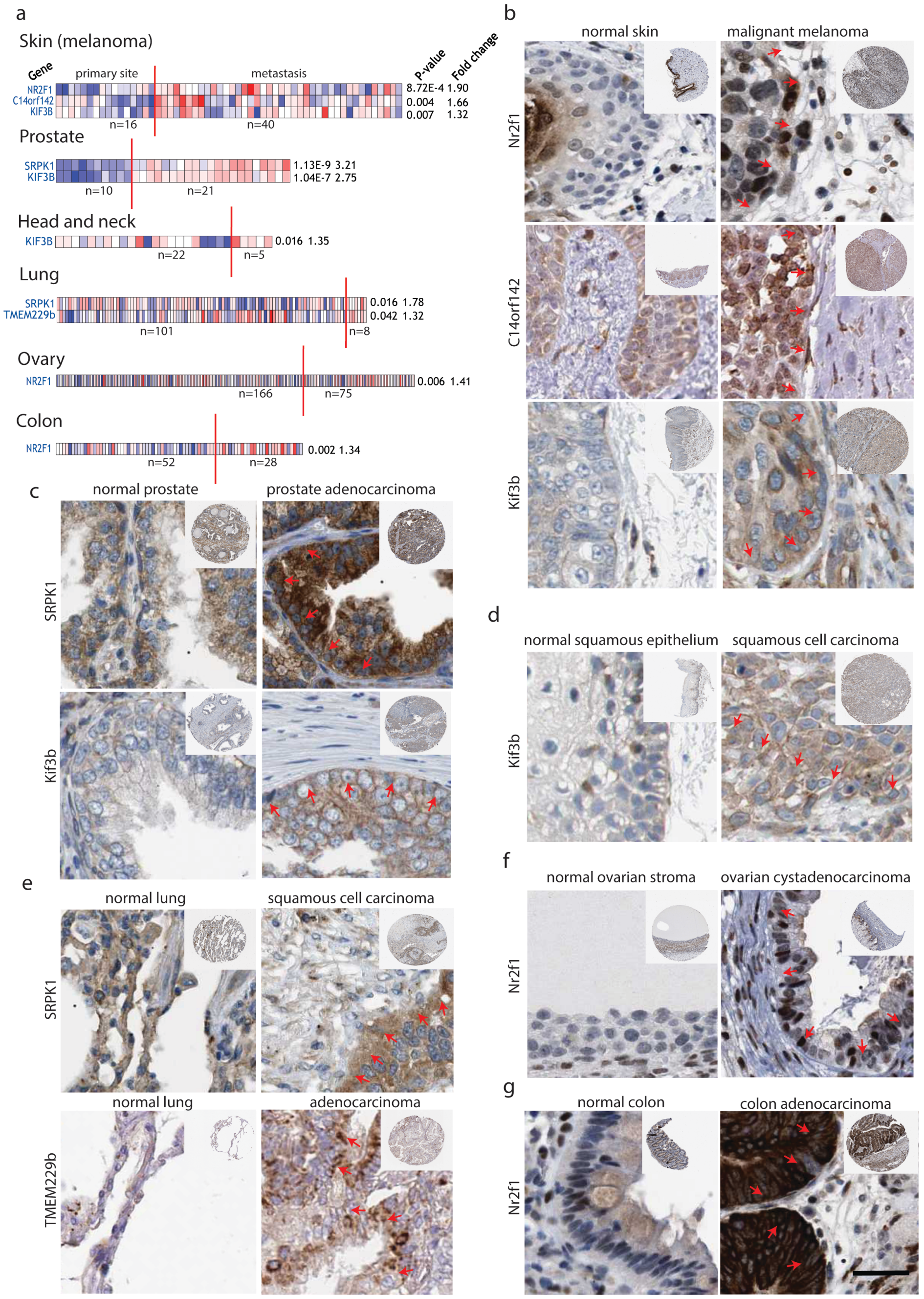

Supplementary Figure 9. Elevated expression of screen-identified genes correlates with cancer cell invasive and metastatic behavior in human cancers. (a) Expression of selected screen hits in metastatic sites versus primary tumors in skin, prostate, head and neck, lung, ovary and colon cancers. In all cases, expression of these genes is significantly elevated in metastases. **(b)** Immunohistochemical analysis of NR2F1, C14orf142 and KIF3B in skin (melanoma) cancer. **c)** Immunohistochemical analysis of SRPK1 and KIF3B in prostate cancer. **(d)**Immunohistochemical analysis of KIF3B in head and neck (squamous cell carcinoma) cancer. **(e)** Immunohistochemical analysis of SRPK1 and TMEM229B in lung cancer. **(f)** Immunohistochemical analysis of NR2F1 expression in ovarian cancer. **(g)** Immunohistochemical analysis of NR2F1 expression in colon cancer. Tumor invasive front in **(c)** to **g)** is indicated by red arrows. Statistical significance was determined using Oncomine built in t -test. Scale bar = 100µm.
