# Supplementary material for: Quantitative in vivo whole genome motility screen reveals novel therapeutic targets to block cancer metastasis
Source: Nat Commun. 2018 Jun 14;9:2343. doi: 10.1038/s41467-018-04743-2 (PMC6002534; doi:10.1038/s41467-018-04743-2)
Supplement: Supplementary file 3 — Description of Additional Supplementary Files [file 41467_2018_4743_MOESM3_ESM.pdf]

Description of Additional Supplementary Files

File Name: Supplementary Data 1
Description: Summary of screen hits. List of shRNAs identified in each clone isolated in the screen, with sequence read numbers, sorted by mean C.I. index.

File Name: Supplementary Movie 1
Description: KIF3B, SRPK1 and NR2F1 are required for productive cancer cell motility. Timelapse intravital imaging sequence showing cancer cell motility in metastatic lesions in vivo formed by control (scramble) shRNA transduced or KIF3B, SRPK1 and NR2F1 knockdown HEp3 cells over 7 hours, 45 minutes. Timestamp is in top left corner; 15 min/ frame, 30 frames. Scale bar = 100 µm.

File Name: Supplementary Movie 2
Description: KIF3B, SRPK1 and NR2F1 are required for invasion at the tumor front. Timelapse intravital imaging sequence showing cancer cell motility at the invasive fronts of primary tumors formed by control (scramble) shRNA transduced or KIF3B, SRPK1 and NR2F1 knockdown HEp3 cells over 7 hours, 15 minutes. Timestamp is in top left corner; 15 min/ frame, 30 frames. Scale bar = 100 µm.

File Name: Supplementary Movie 3
Description: Down-regulation of KIF3B gene expression limits HEp3 cancer cell protrusion lifetime and engagement with collagen matrix. Time-lapse intravital two photon (SHG) imaging sequence showing HEp3 cancer cell behavior within the metastatic lesions formed by control (scramble) shRNA transduced or KIF3B knockdown HEp3 cells over 1 hour over 1h. Timestamp is in top left corner; 3 min/ frame, 20 frames. Scale bar = 100 µm.

File Name: Supplementary Movie 4
Description: KIF3B is required for HEp3 cancer cell interaction with the collagen fiber network within metastatic lesions. Time-lapse intravital two-photon imaging (SHG) sequence showing HEp3 cancer cell behavior within the metastatic lesions formed by control (scramble) shRNA transduced or KIF3B knockdown HEp3 cells over 2.5 hours. Timestamp is in top left corner; 10 min/ frame, 20 frames. Scale bar = 100 µm.

File Name: Supplementary Movie 5
Description: KIF3B is required for HEp3 cancer cell-induced collagen fiber alignment at the tumor front. Time-lapse intravital two-photon imaging (SHG) sequence showing cancer cell motility at the invasive fronts of primary tumors formed by control (scramble) shRNA transduced or KIF3B knockdown HEp3 cells over 1.5 hours. Timestamp is in top left corner; 10 min/ frame, 16 frames. Scale bar = 100 µm.
